# Assessing the metastatic potential of circulating tumor cells using an organ-on-chip model

**DOI:** 10.3389/fbioe.2024.1457884

**Published:** 2024-10-08

**Authors:** Karin F. Schmid, Soheila Zeinali, Susanne K. Moser, Christelle Dubey, Sabine Schneider, Haibin Deng, Simon Haefliger, Thomas M. Marti, Olivier T. Guenat

**Affiliations:** ^1^ Organs-on-chip Technologies Laboratory, ARTORG Center, University of Bern, Bern, Switzerland; ^2^ Graduate School of Cellular and Biomedical Sciences, University of Bern, Bern, Switzerland; ^3^ Department of General Thoracic Surgery, Inselspital, Bern University Hospital, University of Bern, Bern, Switzerland; ^4^ Department of BioMedical Research, University of Bern, Bern, Switzerland; ^5^ Department of Medical Oncology, Inselspital, Bern University Hospital, University of Bern, Bern, Switzerland; ^6^ Department of Pulmonary Medicine, Inselspital, Bern University Hospital, University of Bern, Bern, Switzerland

**Keywords:** microvasculature-on-chip, metastasis, extravasation, A549 subclones, EMT, mesenchymal phenotype, epithelial phenotype, VEGF

## Abstract

Metastatic lung cancer remains a leading cause of death worldwide, with its intricate metastatic cascade posing significant challenges to researchers and clinicians. Despite substantial progress in understanding this cascade, many aspects remain elusive. Microfluidic-based vasculature-on-chip models have emerged as powerful tools in cancer research, enabling the simulation of specific stages of tumor progression. In this study, we investigate the extravasation behaviors of A549 lung cancer cell subpopulations, revealing distinct differences based on their phenotypes. Our results show that holoclones, which exhibit an epithelial phenotype, do not undergo extravasation. In contrast, paraclones, characterized by a mesenchymal phenotype, demonstrate a notable capacity for extravasation. Furthermore, we observed that paraclones migrate significantly faster than holoclones within the microfluidic model. Importantly, we found that the depletion of vascular endothelial growth factor (VEGF) effectively inhibits the extravasation of paraclones. These findings highlight the utility of microfluidic-based models in replicating key aspects of the metastatic cascade. The insights gained from this study underscore the potential of these models to advance precision medicine by facilitating the assessment of patient-specific cancer cell dynamics and drug responses. This approach could lead to improved strategies for predicting metastatic risk and tailoring personalized cancer therapies, potentially involving the sampling of cancer cells from patients during tumor resection or biopsies.

## 1 Introduction

Lung cancer is the leading cause of cancer-associated mortality worldwide, with non-small cell lung cancer (NSCLC) accounting for 80%–85% of all lung cancers (World Health Organization (WHO)). The high fatality rate is mainly due to the advanced stage at which most patients are diagnosed. By this point, the cancer has often metastasized, to other parts of the body, such as the bones, brain, and liver (Tamura et al., 2015; Siegel et al., 2021). Metastasis is an intricate process in which cancer cells escape from the primary tumor, intravasate into the bloodstream or lymphatic system, and ultimately get lodged in small capillaries at distant locations, where they extravasate to form secondary tumors (Lambert et al., 2024). Cancer cells evading primary tumors have a remarkable ability to adapt during the metastatic journey. For instance, circulating tumor cells (CTCs) can undergo epithelial-to-mesenchymal transition (EMT), adopt a regenerative progenitor-like phenotype, enter dormancy, evade immune surveillance, and interact with organ-specific niches (Celià-Terrassa and Kang, 2016; Massagué and Ganesh, 2021; Bakir et al., 2020). In the EMT process, cells shed their epithelial features and acquire mesenchymal characteristics. The role of EMT in cancer metastasis has been widely discussed as both phenotypes, epithelial and mesenchymal, play an essential role during tumor initiation, stemness, therapy resistance, invasion, and migration capacities (Roche, 2018; Shibue and Weinberg, 2017; Nieto et al., 2016; Pastushenko and Blanpain, 2019). Underlying mechanisms of EMT are described to be complex and based on diverse origins such as genetic mutations, epigenetic alterations, miRNA, biochemical or mechanical cues (Kalluri and Weinberg, 2009; Zhao and Lv, 2023; Pan et al., 2021). This adaptability is demonstrated by the presence of different subcolonies that derive from the parental NSCLC cell line A549; holoclones, meroclones, and paraclones. Holoclonal A549 cells give rise to round packed colonies and exhibit an epithelial and stem-cell-like phenotype that features the highest tumor initiation capacity. Paraclonal A549 cells are loosly packed and exhibit a mesenchymal-like phenotype associated with increased resistance to therapy and high migration capacity. Meroclonal cells, however, feature an intermediate phenotype. Xenograft tumor formation capacity is significantly higher in holoclonal cells than in paraclonal A549 cells (Tièche et al., 2019).

*In vitro* and *in vivo* methods have been established to better understand the underlying mechanisms of metastasis and address the inherent complexities of the metastatic process. Standard *in vitro* methods are often simple, fast, and reproducible but mostly limited to two-dimensional or three-dimensional assays based on chemotaxis or the Boyden-chamber principle and lack the tumor microenvironment. *In vivo* methods allow the crosstalk between cancer cells and the organisms based on genetically engineered or transplant model organisms (syngeneic animals, patient- or cell line-derived xenografts) but are time-consuming, expensive and associated with ethical concerns (Bouchalova and Bouchal, 2022). In the past few years, microfluidic-based vascular models, have successfully replicated specific stages of the metastatic process (Brooks et al., 2024; Kim et al., 2022) or simulated various aspects of the tumor microenvironment (Jung et al., 2022), such as the role of mural cells in tumor vessel permeability (Bichsel et al., 2017). During the metastasis, only a few CTCs evade the immune system and withstand the shear stress or anoikis within the bloodstream (Leone et al., 2018; Mitchell and King, 2013; Han et al., 2021). Microfluidic-based vascular models can recapitulate the dynamic multicellular interactions between CTCs and blood vessels, thus demonstrating their significance in cancer research. These models are either based on the self-assembly of endothelial cells and stromal cells or utilized patterned lumen seeded with endothelial cells (Haase and Kamm, 2017). Organotypic microfluidic assays have shown that breast cancer cells do not exhibit extravasation dynamics into the muscle microenvironment, but do so into bone, through the interplay of breast cancer cell receptor CXCR2 and the chemokine CXCL5 secreted by the bone (Bersini et al., 2014; Jeon et al., 2015). Crippa et al. recreated an early metastatic niche by perfusion of CTCs together with platelets and neutrophils in a vascularized 3D microvascular environment, which showed increased extravasation dynamics due to the upregulation of EMT markers in the presence of the immune cells. Further, drug testing with eptifibatide, an integrin β3 inhibitor, reduced the expression of EMT markers in cancers and decreased cancer cell adhesion as well as invasion (Crippa et al., 2021). Boussommier-Calleja et al. reported the monocyte-mediated impact on cancer cell extravasation showing that undifferentiated monocytes reduce cancer cell extravasation (Boussommier-Calleja et al., 2019). In addition, Offeddu et al. demonstrated that tumor and vascular glycocalyx play a crucial role in tumor cell extravasation, providing evidence that tumor cells repurpose glycocalyx to promote adhesive interactions and cancer progression (Offeddu et al., 2021).

In contrast to earlier studies, the aim of this research is to evaluate the behavior of lung cancer cell subpopulations with different phenotypes within a functional microvascular network (µVN). Specifically, we focus on holoclones and paraclones, two A549 lung adenocarcinoma cell subpopulations characterized by epithelial and mesenchymal phenotypes, respectively. By introducing these cancer cells into a self-assembled microvasculature network, we aim to assess their extravasation potential and migration velocities. Additionally, we investigate the impact of extravasation capacity upon VEGF depletion in the cell culture, simulating the effects of anti-VEGF therapeutic drugs. This study is novel in its comprehensive approach to examining the differential behavior of lung cancer subpopulations in a dynamic vascular environment, providing valuable insights into their metastatic capabilities and potential therapeutic responses.

## 2 Material and methods

### 2.1 Chip design and fabrication

The design and fabrication of the microvasculature-on-chip (µVN-on-Chip) were reported earlier (Bichsel et al., 2015). Briefly, the design includes a 2 mm in diameter circular central chamber and two adjacent microchannels for cell culture medium and for cancer cell loading. They are separated by a series of trapezoidal micropillars aimed at maintaining the hydrogel within the central chamber by surface tension. The microfluidic chips were produced by polydimethylsiloxane (PDMS) replica molding on a SU-8-coated Si wafer. Briefly, PDMS (Sylgard™ 184 Elastomer, Dow Corning, Midland, MI, U.S.) was mixed in a 10:1 ratio with the curing agent and cured at 60 °C for at least 6 hours. Prepared chips were treated with oxygen plasma prior to bonding to glass microscopy slides.

### 2.2 Cell culture

Primary human umbilical vein endothelial cells (HUVECs) were purchased from Gibco (Thermo Fisher Scientific, Waltham, MA, U.S.) and cultured in endothelial growth medium 2 (Cat. #CC-3162, Lonza, Basel, Switzerland). RFP-positive primary human lung microvascular endothelial cells (VeraVecs, Angiocrine Bioscience, San Diego, CA, U.S.) were cultured in microvascular endothelial growth medium 2 (Cat. #CC3202, Lonza, Basel, Switzerland). Normal human lung fibroblasts (NHLFs) were obtained from Lonza (Basel, Switzerland) and cultured in Ham’s F-12K (Kaighn’s) cell culture medium (Cat. #21127022, Thermo Fisher Scientific, Waltham, MA, U.S.) supplemented with 10% fetal bovine serum (Cat. #F7524, Sigma-Aldrich, St. Louis, MO, U.S.) and 1% Penicillin/Streptomycin (Cat. #P4333, Sigma-Aldrich, St. Louis, MO, U.S.). The NSCLC cell line A549 (CCL-185) was purchased from the American Type Culture Collection (ATCC, Manassas, VA, U.S.). The cell lines paraclone and holoclone were obtained from parental A549 as described previously (Tièche et al., 2019) and cultured in Dulbecco’s Modified Eagle Medium F12 (DMEM/F12) Nutrient Mixture Ham (Cat. #21331020, Thermo Fisher Scientific, Waltham, MA, U.S.) supplemented with 10% fetal bovine serum (Cat. #F7524, Sigma-Aldrich, St. Louis, MO, U.S.), 1% Penicillin/Streptomycin (Cat. #P4333, Sigma-Aldrich, St. Louis, MO, U.S.), and 1 L-glutamine (Cat. #5030024, Invitrogen, Waltham, MA, U.S.). Subtype-specific colonies were identified based on their ability to form colonies (holoclones) and subsequently separated by cloning cylinders to establish subpopulations. All cells were maintained at 37°C and 5% CO_2_ in a humidified incubator. Harvesting of cells was performed by treatment with TrypLE Express Enzyme 1X (Cat. # 12604039, Thermo Fisher Scientific, Waltham, MA, U.S.), and cell numbers were determined using a hemocytometer and 0.1% trypan blue for dead cell exclusion.

### 2.3 Lentiviral infection of A549 subpopulations

The lentiviral vectors were produced according to a previously published protocol with a few modifications (Beronja et al., 2010). In detail, VSV-G pseudotyped lentivirus was produced by transfection of 293T cells (Invitrogen, Waltham, MA, U.S.) using Lipofectamine 2000, 9 µg packaging plasmid psPAX2 (Cat. #12260, Addgene, Watertown, MA, U.S.), 0.9 µg envelope plasmid pCAG-VSVG (Cat. #35616, Addgene, Watertown, MA, U.S.), 9 µg LV-GFP (Cat. #25999, Addgene, Watertown, MA, U.S.) or LV-RFP (Cat. #26001, Addgene, Watertown, MA, U.S.) in 225 µL Opti-MEM I (Cat. #31985062, Thermo Fisher Scientific, Waltham, MA, U.S.) mixed with 90 µL Opti-MEM containing 54 µL Lipofectamine 2000 (Cat. 11668019, Thermo Fisher Scientific, Waltham, MA, U.S.). Medium was exchanged after 18 h of incubation. Virus supernatant was collected 24 h and 48 h later. 500 μL of virus supernatant was used to transduce A549 subpopulations ^8^ in the presence of 8 μg/mL polybrene for 72 h. Cells were then flow-sorted twice for GFP or RFP-positive populations using BD FACS ARIA I (BD Biosciences, Franklin Lakes, NY, U.S.).

### 2.4 FACS

For the analysis by flow cytometry, both A549 subpopulations were cultured for 7 days. After washing in phosphate-buffered saline (PBS, Cat. #D8537, Sigma-Aldrich, St. Louis, MO, U.S.), cells were either cultivated in their standard cell culture medium as described above or cultivated in microvasculature cell culture medium (EGM2) for 24 h. Cells were cultivated at 37°C and 5% CO_2_ in a humidified incubator and harvested as described previously with few adaptations (Deng et al., 2022). In detail, for the extracellular staining cells were washed with PBS, about 500,000 cells were resuspended in 100 µL of FACS buffer (2% FBS/PBS) supplemented with 20 µL of human TruStain FcX™ Fc Receptor Binding Inhibitor Functional Grade Monoclonal Antibody (Cat. #14–9161–73, eBioscience, San Diego, CA, U.S.) and incubated for 20 min at RT. 1 mL FACS buffer was added to stop the reaction and cells were washed, followed by centrifugation at 400 g for 5 min. After discarding the supernatant, cells were incubated in FACS buffer containing the extracellular mouse anti-human EpCAM-PE-Cyanine7 (1.5 µL/100 µL) (Cat. #25–9326–42, eBioscience, San Diego, CA, U.S.), mouse anti-human CD90-BV421 (3 µL/100 µL) (Cat. #328122, BioLegend, San Diego, CA, U.S.) and LIVE/DEAD™ Fixable Near-IR Dead Cell Stain Kit (Cat. #L10119, Thermo Fisher Scientific, Waltham, MA, U.S.) for 30 min on ice (protected from light). Cells were washed in FACS buffer for 5 min and then centrifuged at 400 *g* for 5 min at RT. The cells were resuspended in 0.5 mL of FACS buffer. All samples were measured on a BD Bioscience LSR2 flow cytometer (BD Biosciences, Franklin Lakes, NY, U.S.), and 10,000 events were recorded. FlowJo V10 software (Tree Star, Inc., Ashland, OR, U.S., RRID: SCR_008520) was used to analyze. fcs files.

### 2.5 Chip loading and maintenance

Both endothelial cells and lung fibroblasts were resuspended in 2 U/mL thrombin from bovine plasma (Cat. #T4648, Sigma-Aldrich, St. Louis, MO, U.S.) in EGM2 at a final concentration of 4 × 10^7^ cells/mL (HUVECs) and 1 × 10^7^ cells/mL (NHLFs), respectively. For co-culture, HUVECs and NHLFs were mixed with 10 mg/mL fibrinogen from bovine plasma (Cat. #F8630, Sigma-Aldrich, St. Louis, MO, U.S.) in PBS(−) (Thermo Fisher Scientific, Waltham, MA, U.S.), followed by immediate pipetting of the cell suspension into the central chamber of the chip. After fibrin polymerization, EGM2 was added to the microchannels as a cell culture medium. The chips were kept at 37°C and 5% CO2 in the incubator in a closed Petri dish, together with a wet tissue for humidification and maintained for 7 days. Medium was exchanged every 24 h. The EGM2 cell culture medium was supplemented with recombinant human VEGF (165) IS (Cat. #130–109–381, Miltenyi Biotec, Bergisch Gladbach, Germany) at 37.5 ng/mL in 0.1% BSA in PBS, sphingosine-1-phosphate (Cat. #S9666, Sigma-Aldrich, St. Louis, MO, U.S.) at 250 nM in 95% DMSO/5% HCl (1M), PMA (Cat. #1585, Sigma-Aldrich, St. Louis, MO, U.S.) at 10 μg/mL DMSO in MiliQ, rhFGFb (Cat. #100–26, Peprotec, Thermo Fisher Scientific, Waltham, MA, U.S.) at 37.5 ng/mL in 0.1% BSA in PBS, recombinant human MCP-1 (Cat. # 11343386, Immunotools, Friesoythe, Germany) 37.5 ng/mL in 0.1% BSA in PBS, and recombinant human HGF (Cat. #294-HG-005, Peprotec, Thermo Fisher Scientific, Waltham, MA, U.S.) at 37.5 ng/mL in 0.1% BSA in PBS from day one until day four to enhance vasculogenesis.

### 2.6 Extravasation assay

**FIGURE 1:**
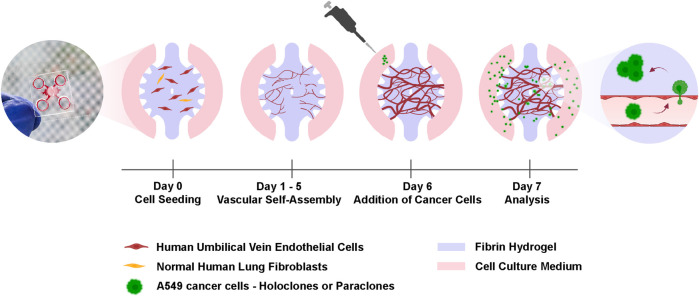
Experimental setup for the cancer cell extravasation-on-chip. Endothelial cells (HUVECs) and stromal cells (NHLFs) are co-cultured in fibrin hydrogel to form an interconnected and perfusable microvasculature within 6 days in the central chamber of a microfluidic chip. On day 6, A549 subclones, holoclones or paraclones, are added to the adjacent microchannels of the central chamber. After 24 h cancer cell extravasation is assessed by live-cell imaging and immunostaining. Created with BioRender.com.

To study cancer cell extravasation, A549 subpopulations, holoclones and paraclones, were added at 2 × 10^5^ cells/mL in EGM2 on day six to the microchannel and incubated for 24 h as shown in Figure 1. All reservoirs were emptied before the addition of 70 µL cancer cell suspension in the microvasculature. The resulting hydrostatic pressure difference across the chip induced a transient flow that directed the cancer cells to enter the vascular lumen through vessel openings. Once cancer cells reached the microvasculature after a few seconds, endothelial growth medium was added to the opposite reservoirs, resulting in a static and equilibrated system. The extravasation efficiency was assessed after confocal imaging of the entire microvasculature network. Images were reconstructed in 3D to quantify extravasated cancer cells (Chen et al., 2017).

### 2.7 VEGF depletion in the µVN-on-chip

A VEGF-free medium (EGM2 w/o VEGF) was used to treat the microvasculature network after mature network formation on day five to prevent VEGF from influencing extravasation results. Mesenchymal-like paraclones were resuspended in EGM2 w/o VEGF and added on day 6 for the extravasation assay.

### 2.8 Permeability assay

To characterize the vascular permeability after cancer cell loading, 70 kDa rhodamine isothiocyanate (RITC) - conjugated dextran (Cat. #R9379, Sigma-Aldrich, St. Louis, MO, U.S.) was added to one reservoir at a concentration of 1 mg/mL in PBS shortly after starting image acquisition. Time-lapse images were acquired every 10 s for 3 min using the M7000 EVOS microscope (Thermo Fisher Scientific, Waltham, MA, U.S.). The permeability coefficient was calculated as previously described (Chrobak et al., 2006). Seven to ten regions of interest were quantified per microvasculature network. To remove the confounding factor of having only local effects on the vascular barrier by cancer cells, additional experiments with A549 conditioned medium were performed. Therefore, DMEM/F12+++ was replaced by EGM2 in the cell culture flasks of A549 subpopulations. Conditioned medium was collected after 24 h and added to the µVN-on-chip on day six.

### 2.9 Immunostaining and image acquisition

For immunofluorescence staining, the microvasculature network was fixed using paraformaldehyde (PFA; 4% w/v in PBS) for 15 min, washed 3x with PBS, permeabilized for 10 min with 0.1% Triton X-100 (Cat. #X100, Sigma-Aldrich, St. Louis, MO, U.S.) and blocked for 1 hour with 2% BSA (Cat. #A9418, Sigma-Aldrich, St. Louis, MO, U.S.) in PBS. Samples were incubated for 24 h with a primary polyclonal goat IgG antibody to the endothelial-specific marker VE-Cadherin (Cat. #AF938-SP, R&D systems, Minneapolis, MN, U.S.) diluted 1:200 in 2% BSA in PBS. As secondary antibody, a donkey anti-Goat IgG conjugated with 546 Alexa Fluor diluted 1:500 (Cat. #A11056, MolecularProbes, Eugene, OR, U.S.) was added together with Acti-stain phalloidin 670 (#Cat. PHDN1, Denver, CO, U.S.) diluted 1:150, and Hoechst 33342 (Cat. # 14533, Sigma-Aldrich, St. Louis, MO, U.S.) diluted 1:1000 in 2% BSA in PBS and incubated for 24 h at 4°C. Z-stack images were acquired using the ×4 and ×10 objectives of the Zeiss laser scanning microscope 710 (Carl Zeiss Microscopy, Thornwood, NY, U.S.). 3D surface rendering was performed with Imaris V9.9.1 (Oxford Instruments, Abington, U.K.).

### 2.10 Time-lapse observation and analysis

For time-lapse observation of the extravasation dynamics of the A549 subpopulations in the microvasculature network, chips were imaged every 30–60 min for 12 h with the Nipkow spinning disk confocal Yokogawa CQ-1 microscope (Yokogawa Denki K.K., Musashino, Japan). After image acquisition, GFP-labeled A549 subpopulations were manually tracked using the open-source image analysis software FIJI (Schindelin et al., 2012). Therefore, selected cancer cells were followed within the vasculature and in the surrounding hydrogel. Obtained results were analyzed for migration velocity and accumulated distance using the open-source Chemotaxis and Migration Tool V2.0 (ibidi, Gräfelfing, Germany). Cell migration trajectories from XY coordinates were performed using R Statistical Software V4.1.2. (R Core Team 2021). Therefore, obtained XY coordinates were converted from pixels to micrometers, and the initial position of each track was subtracted for each track (X = X–first(X), Y = Y–first(Y)) to start at (0,0) for each track. The R analysis pipeline included open-source packages including dplyr (Wickham et al.) as well as ggplot (Wickham, 2016).

### 2.11 RT-qPCR

To assess the effect of A549 cancer cell conditioned medium on endothelial gene expression, HUVECs were seeded at a 40 × 10^6^ cells/mL in a 10 µL fibrin hydrogel droplet on a 96-well plate. After 3 days in EGM2 cell culture medium, HUVECs were exposed for 24 h to A549 conditioned medium of holoclones or paraclones, respectively. Microvasculature droplets were lysed with TriReagent (Cat. #R2050-1-50, Zymo Research, Irvine, CA, U.S.) followed by RNA isolation with Direct-zol RNA MicroPrep (Cat. #R2062, Zymo Research, Irvine, CA, U.S.) and reserve transcription using the iScript Kit (Cat. # 1708891 BioRAD, Hercules, CA, U.S.). Quantitative PCR was performed in duplicates with Sensifast SYBR low ROX kit (Cat. #BIO94005, Labgene Scientific, Châtel-Saint-Denis, CH) using QuantStudio 3 (ThermoFisher Scientific, Waltham, MA, U.S.). For analysis, *ACTB*, *GAPDH,* and *HPRT1* were used as housekeeping genes. Obtained mean ΔCq values were used to calculate the relative gene expression levels of VE-cadherin using the 2^−ΔΔCq^ method (Livak and Schmittgen, 2001). Primers are listed in Supplementary Table S1.

### 2.12 Statistical analysis

Statistical analyses were performed in GraphPad Prism, v.10.0.3 (GraphPad Software Inc., San Diego, CA, U.S.) using statistical tests indicated in the figure legends. Normality of the data was checked by a Shapiro-Wilk test. For all experiments, *p* < 0.05 was considered statistically significant. Values are reported as mean±SD.

### 2.13 Language editing assistance

The grammar and spelling of the manuscript were checked by ChatGPT, v3.5 (ChatGPT, OpenAI Inc, CA, United States) to ensure clarity and correctness without affecting the scientific content.

## 3 Results

### 3.1 A549 holoclones and paraclones maintained their phenotype in endothelial growth medium

**FIGURE 2:**
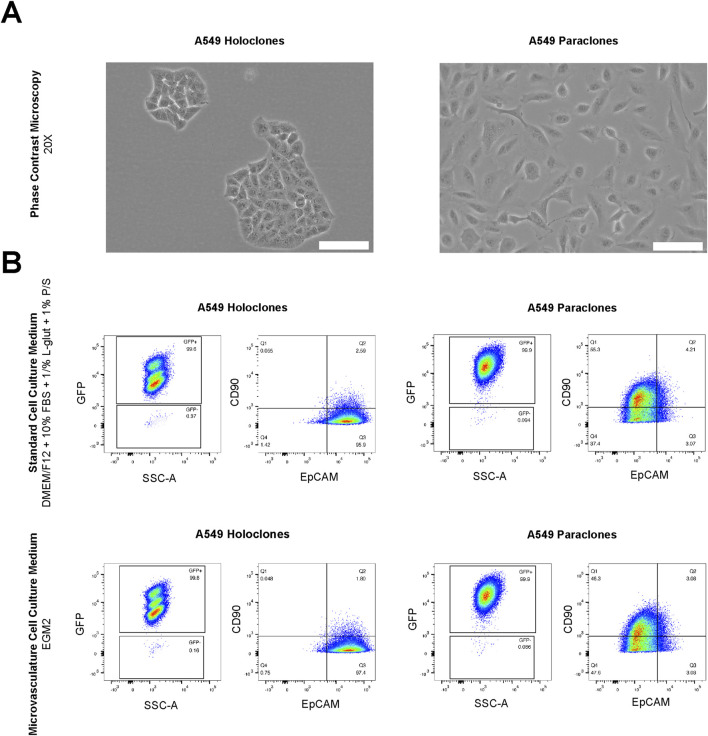
Morphological and FACS analysis of A549 subclones. **(A)** A549 holoclones and paraclones are shown by phase contrast microscopy. Scale bar: 100 µm. **(B)** Analysis by flow cytometry in standard cell culture medium (DMEM/F12 with supplements) and microvasculature cell culture medium (EGM2) show no differences in their expression phenotype. GFP positive holoclones and paraclones feature EpCAM+/CD90-and EpCAM-/CD90+, respectively.

First, we assessed whether the subtype-specific characteristics of A549 subpopulations are influenced by differences in culture conditions, in particular the physiological medium used to growth the microvasculature. In detail, the cultures of both A549 subpopulations, holoclones and paraclones, exhibited the subtype-specific cellular morphology previously described (Tièche et al., 2019) as observed by phase contrast microscopy (Figure 2A). Epithelial-like holoclones formed round colonies with distinct borders of small, packed cells, whereas mesenchymal-like paraclones were elongated without distinct colony formation. As A549 subpopulations present phenotypic plasticity over time (Tièche et al., 2019), both A549 subpopulations were analyzed by flow cytometry. This assessment was conducted following a 24-h exposure to standard cell culture medium (DMEM/F12 with supplements) or endothelial growth medium (EGM2), the medium used for culture A549 subpopulations during expansion or testing in µVN-on-chip, respectively. Gating strategies illustrated in Figure 2B were used to interrogate GFP-positive cells for the epithelial and mesenchymal markers EpCAM and CD90, respectively. GFP^+^ A549 holoclones and paraclones showed no discernible alteration in their characteristic expression profiles when cultured in standard cell culture medium compared to the endothelial growth medium. GFP^+^ holoclones consistently displayed the characteristic EpCAM^+^/CD90^-^ phenotype. Three subpopulations were identified within GFP^+^ holoclones, each exhibiting consistent EpCAM^+^/CD90^-^ patterns (Supplementary Figure S1). In contrast, GFP^+^ paraclones demonstrated a contrasting profile with EpCAM^−^/CD90^+^. These findings underscore the suitability of our experimental setting used in our µVN-on-chip system to explore the extravasation properties of phenotype-specific cancer cells.

### 3.2 A549 subpopulations showed distinct extravasation dynamics

**FIGURE 3:**
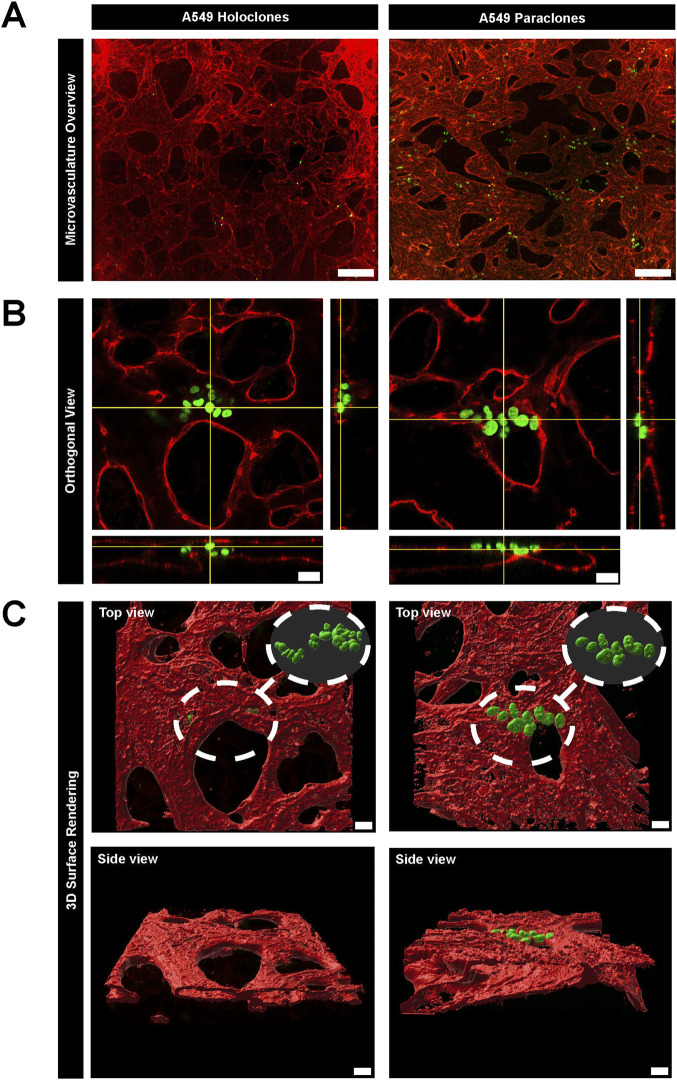
Extravasation dynamics of two subpopulations of A549 cancer cells in a µVN-on-chip. **(A)** Representative images show GFP + A549 holoclones (left column) and paraclones (right column) within the microvasculature. The maximum Z projection of the microvasculature overview show that holoclones remain inside the microvasculature, whereas paraclones extravasate into the surrounding hydrogel. VE-cadherin = red, A549 subclones = green, scale bar = 200 µm. **(B)** Orthogonal views show cancer cells located within the microvasculature network. Holoclones remain within the endothelial lining marked by PECAM1, whereas paraclones extravasate in the hydrogel. PECAM1 = red, A549 subclones = green, scale bar = 20 µm. **(C)**. 3D surface rendering (IMARIS software) illustrate the different extravasation dynamics of both A549 subclones from top and side view. Scale bar = 20 µm.

To study the extravasation dynamics of cancer cells, A549 subpopulations were introduced separately into a perfusable and functional microvasculature, created by self-assembly of primary HUVECs or primary human lung microvascular endothelial cells and NHLFs in the central chamber of the µVN-on-chip (Zeinali et al., 2018). Vessel openings between the micropillars were demonstrated by permeability assay with 70 kDa RITC Dextran (Supplementary Video S1). This setup enabled cancer cells to migrate across the microvasculature using their intrinsic migration capacities. The extravasation dynamics of the cancer cells were assessed after 24h by confocal microscopy based on immunostaining (Figure 3). A549 holoclones remained confined within the microvasculature, whereas A549 paraclones crossed the endothelial barrier and migrated into the surrounding fibrin hydrogel (Figure 3A; Supplementary Table S2). The orthogonal views (Figure 3B) illustrated that A549 holoclones attach to the endothelial lining of the microvasculature without extravasating. In contrast, paraclones were found in the extracellular matrix. A 3D surface rendering clearly showed the difference (Figure 3C). It should be mentioned that the low number of holoclones found in the microvasculature was not due to a difference in cell loading concentration but rather a result of the washing steps during the immunostaining procedure that removed most of them. It is worth noting that the extravasation dynamics of both A549 subpopulations were similar in an organotypic lung microvasculature created with human lung microvascular cells and human lung fibroblasts compared to a microvasculature based on HUVECs and human lung fibroblasts (Supplementary Figure S3).

### 3.3 VEGF depletion prevented A549 paraclone extravasation

**FIGURE 4:**
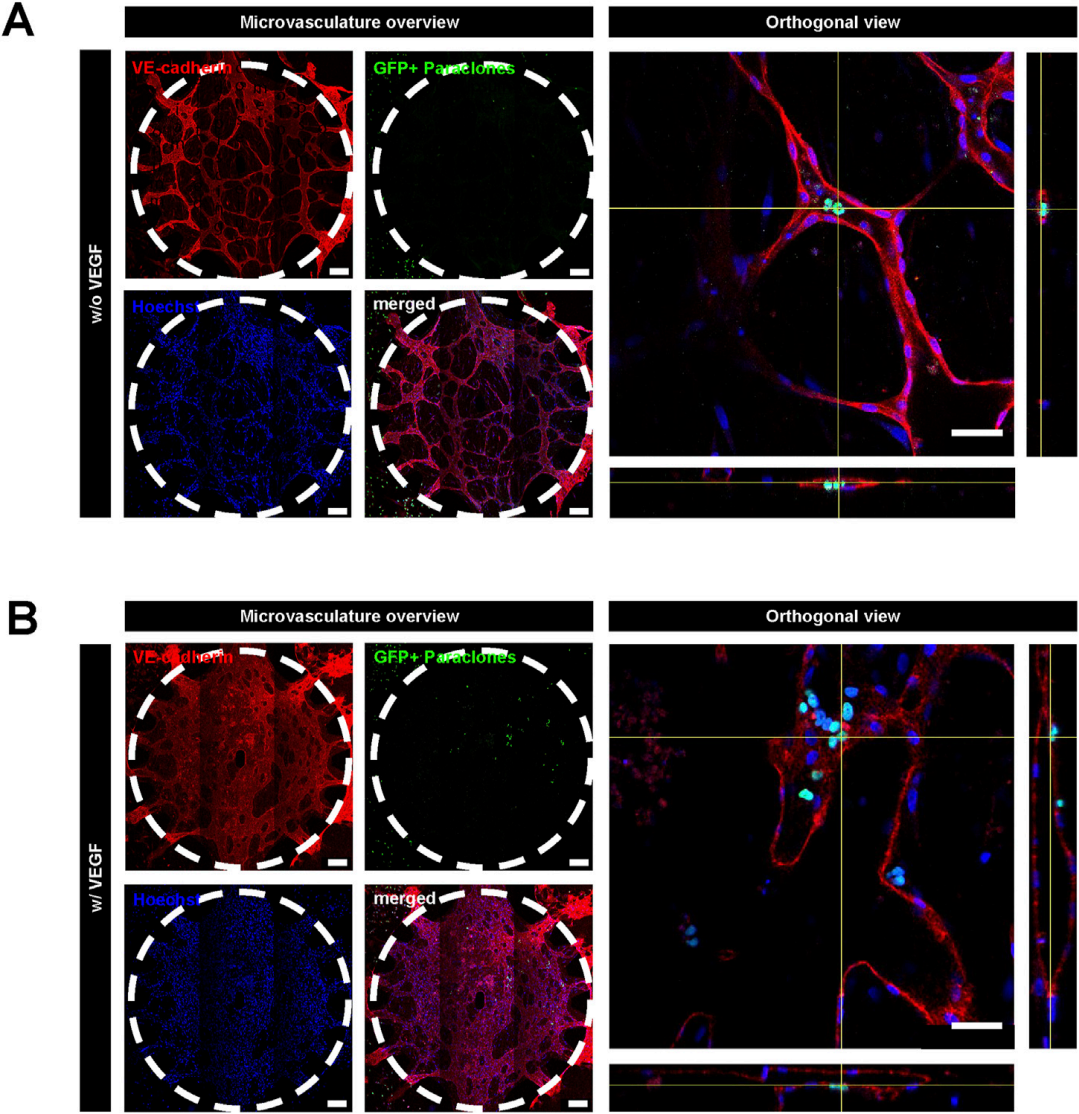
Extravasation assay with A549 paraclones with and without VEGF. After the microvasculature formation on day 5, a cell culture medium without VEGF was used. A549 paraclones were added on day 6 and incubated for 24 h. **(A)** Representative images show no paraclone extravasation when VEGF is absent. **(B)** With VEGF, paraclones enter the microvasculature network and extravasate into the surrounding fibrin hydrogel. Scale bar microvasculature overview: 200 μm, scale bar orthogonal view: 50 µm.

Since EGM2 contains VEGF, which is known to render blood vessels leaky (Ferrara, 2004), we tested whether paraclone extravasation is enhanced by VEGF. For this purpose, we used a medium without VEGF after day 5, after the vasculature had formed. A549 paraclones were suspended in endothelial growth medium w/o VEGF and introduced in the microvasculature (Figure 1). After 24 h, the GFP^+^ paraclones without VEGF remained in the microvasculature network and did not extravasate, in contrast to the paraclones cultured with VEGF (Figure 4). Most paraclones cultured without VEGF were washed away during the immunostaining washing procedure, which explains the low number of GFP^+^ cells trapped in small capillaries in Figure 4A. In contrast, Figure 4B shows extravasated GFP^+^ paraclones (with VEGF). Additional immunostaining and quantification obtained with other µVN-on-chips are illustrated in Supplementary Figure S3; Supplementary Table S3.

### 3.4 Impact of A549 cancer cells on the vascular integrity

**FIGURE 5:**
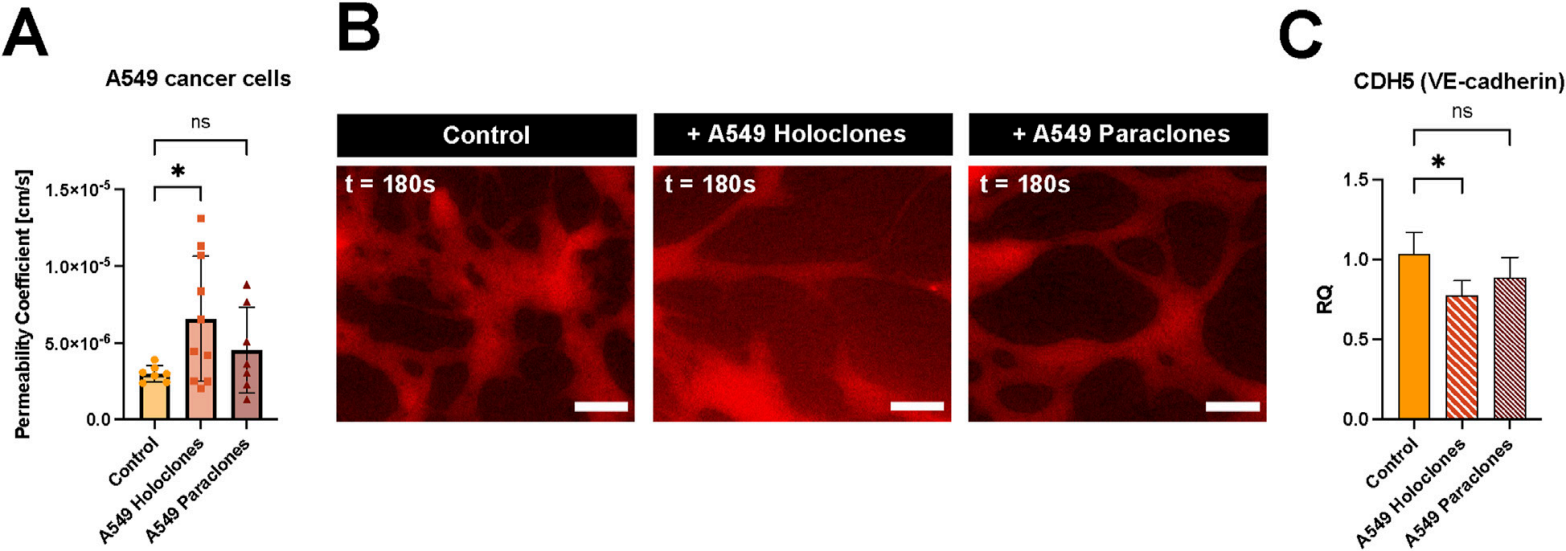
Impact of A549 cancer cells on vascular permeability. **(A)** Vascular permeability was assessed by 70 kDa RITC Dextran perfusion over 3 minutes. A549 subclones, holoclones and paraclones, were resuspended in endothelial medium and added to the µVN-on-chip for 24 h before the permeability assay. Plot: mean permeability (SD), statistical analysis: ordinary one-way ANOVA with Dunnett’s multiple comparison test. N = 7–10 chips per condition, at least three independent experiments. *p < 0.05. **(B)** Relative quantification (RQ) of VE-cadherin gene expression in HUVECs after 24 h exposure to conditioned medium of A549 holoclones and paraclones. Plot: mean permeability (SD), statistical analysis: ordinary one-way ANOVA with Dunnett’s multiple comparison test, n = 4. *p = 0.025. **(C)** Fluorescent images show 70 kDa RITC Dextran (red) diffusion for three conditions: without cancer cells, with holoclones, with paraclones at t = 180 s. Scale bar = 100 µm.

The permeability change of the vascular barrier due to the presence of A549 subpopulations (200,000 cells per mL) was assessed after 24 h incubation in the network. The permeability doubled when holoclones were present in the microvasculature compared to the situation without cancer cells. However, no significant changes were observed when paraclones were added to the network. The calculated permeability coefficient of µVN was 6.57 ( ± 4.07) x 10^–6^ cm/s and 4.54 ( ± 2.79) x 10^–6^ cm/s with A549 holoclones or paraclones, respectively, compared to the control µVN without cancer cells 3.00 ( ± 0.53) x 10^–6^ cm/s (Figure 5A). Figure 5B shows representative images of the dye diffusing from the microvasculature without cancer cells, with holoclones and paraclones. The effect of the conditioned media from A549 subpopulations in EGM2 media on vascular permeability was also investigated. The conditioned media did not affect the permeability after 24 h of exposure. The vascular permeability was found to be 4.15 ( ± 2.63) x 10^–6^ cm/s (paraclones) and 3.28 ( ± 3.81) x 10^–6^ cm/s (holoclones) (Supplementary Figure S4). To investigate the genetic levels of endothelial cells exposed to conditioned medium of A549 subpopulations, qPCR was performed with HUVECs in fibrin cultivated with endothelial growth medium or A549 conditioned medium. The relative quantification (RQ) plot showed a significant decrease in the gene expression profile of VE-cadherin in HUVECs (RQ 0.78 ( ± 0.09)), when exposed with A549 holoclones conditioned medium. However, no significant differences were measured when exposed to A549 paraclones conditioned medium (RQ 0.88 ( ± 0.13)) (Figure 5C). Furthermore, no changes were observed after evaluating the protein expression of VE-cadherin by immunostaining after adding A549 cancer cells or conditioned medium, respectively, to the microvasculature network (Supplementary Figure S5).

### 3.5 High migration kinetics in mesenchymal-like paraclones

**FIGURE 6:**
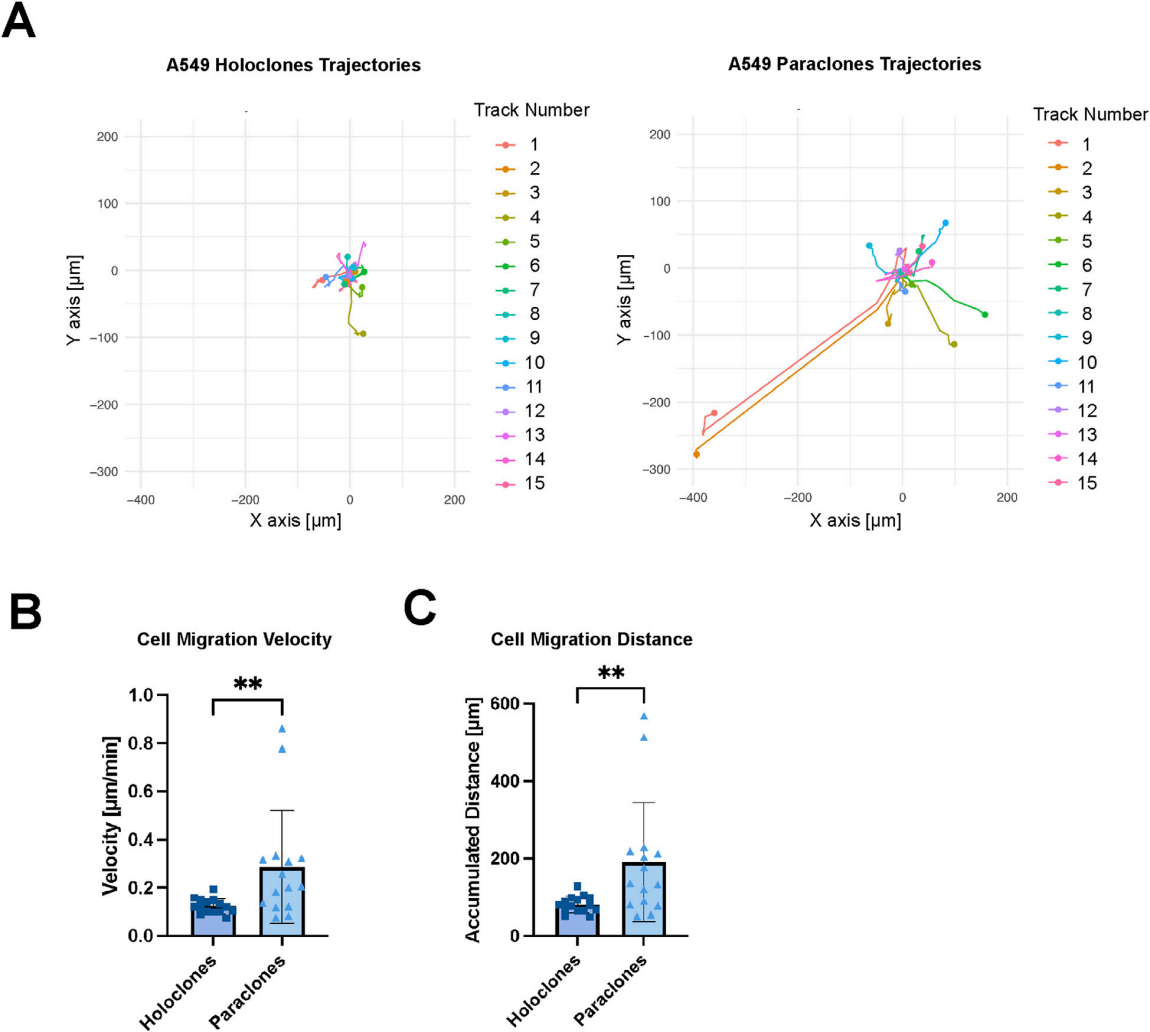
Migration kinetics of A549 subclones. Holoclones and paraclones were introduced through a µVN-on-chip. X/Y coordinates were recorded during live-cell imaging for 12 h. **(A)** Cell migration trajectories are shown for A549 holoclones and paraclones. **(B)** Mean cell migration velocity is shown for A549 holoclones and paraclones. **(C)** The accumulated cell migration distance is shown for both A549 subclones. Statistical analysis: Mann-Whitney U test, n = 15, **p < 0.01. Plot: mean with SD for the individual data points. At least three independent experiments.

Live imaging was performed to evaluate the migration of cancer cells within the microvasculature over 12 h. Mesenchymal-like paraclones exhibited a high migratory pattern and extravasation dynamics (Supplementary Video S2), whereas holoclones displayed a significantly reduced migratory pattern, while remaining within the microvasculature (Supplementary Video S3). In each case, the migration trajectories of 15 cells were recorded (Figure 6). Paraclones migrated for up to hundreds of micrometers in the microvasculature with a calculated cell migration velocity of 0.29 ( ± 0.23) mm/min. In contrast, holoclones were less motile within the vascular space 0.12 ( ± 0.03) µm/min. Paraclones moved significantly faster than holoclones and thus the accumulated distance over 12 h was significantly higher for paraclones 190.9 ( ± 154.3) µm than for holoclones 81.1 ( ± 21.2) µm.

## 4 Discussion

In this work, we demonstrate that specific phenotypes of lung adenocarcinoma subpopulations predispose them to either extravasate through the endothelial barrier of tiny capillaries or remain within these capillaries. While A549 holoclones, characterized by an epithelial phenotype, were identified as drivers of tumor growth (Tièche et al., 2019), we show that A549 paraclones with a mesenchymal-like phenotype extravasate across the endothelial barrier, similar to mesenchymal-like CTCs at distant sites (Di Russo et al., 2024), thereby validating this process *in vitro*.

### 4.1 A549 subpopulations maintain their phenotypes in vasculature medium

Ye et al. have shown that the A549 cancer cell line, a common NSCLC model, contains morphologically distinct subpopulations: holoclones, meroclones, and paraclones (Ye et al., 2011). Tièche et al. performed further characterization to define stemness, tumor initiation, therapy resistance, invasion, migratory capacity, and gene expression profiles of each subpopulation (Tièche et al., 2019). It has been shown that the presence of subpopulations with different EMT phenotypes is not restricted to the A549 adenocarcinoma cell line but also occurs in prostate, pancreatic, breast, uveal, head, and neck cancer cell lines (Li et al., 2008; Tan et al., 2011; Liu et al., 2013; Kalirai et al., 2011; Harper et al., 2007). As phenotypic plasticity of A549 subpopulations has been reported across multiple passages (Tièche et al., 2019), we first analyzed both subpopulations by flow cytometry ^41^. This was done in their standard cell culture medium (DMEM/F12+++) and after 24 h in microvasculature cell culture medium (EGM2) like in our µVN-on-chip system. Round-shaped A549 holoclones were identified as EpCAM^+^/CD90^-^, whereas elongated-shaped paraclones were identified as EpCAM^−^/CD90^+^, as previously reported (Tièche et al., 2019). Cellular stress induced by medium change had no relevant effect on the characteristic expression pattern of both A549 subpopulations within 24 h. Holoclones remained EpCAM^+^/CD90^-^, whereas paraclones remained EpCAM^−^/CD90^+^ as expected in this time frame. Meroclones were not investigated as they present an intermediate phenotype.

### 4.2 CTC phenotype in microvasculature defines their behavior

Microfluidic-based vascular tumor-on-chip models have already been used in several cancer research studies (Kim et al., 2017; Osaki et al., 2018), for example, to recapitulate the site of cancer cell extravasation (Fu et al., 2018). CTCs often arrest in the microvasculature due to physical trapping or by cell-cell or cell-matrix interactions, respectively (Orr and Wang, 2001; Chen et al., 2016; Chambers et al., 2002). The *in vitro* extravasation assay presented in this study is based on our previously reported µVN-on-chip (Bichsel et al., 2015; Zeinali et al., 2018), which replicates a functional microvasculature network. The microvascular model is based on the self-assembly of endothelial cells and fibroblasts, which form an interconnected network with tortuous, variable morphology and differing vessel dimensions, mirroring characteristics *in vivo* (Zeinali et al., 2018). In agreement with earlier studies, paracrine signaling by co-culture of endothelial cells with stromal cells together with exogenous proangiogenic growth factors such as VEGF led to vessel diameter range between 1 and 100 µm in our system (Whisler et al., 2014; Tan et al., 2022). A549 subpopulations were introduced into the capillary network to interact with this microenvironment, similar to their behavior at a secondary site. When CTCs arrive in the pre-metastatic niche *in vivo*, the vasculature undergoes remodeling followed by altering of the extracellular matrix by depositing new components, induced by activated stromal fibroblasts (Peinado et al., 2017). We demonstrated that holoclones and paraclones differ in their extravasation dynamics and, thus, in the metastatic risk they represent. Live-cell imaging analysis reveals that epithelial-like holoclones migrate at low velocity and remain inside the vasculature lumen, whereas mesenchymal-like paraclones express high migration velocity and extravasate to the surrounding hydrogel. It was previously demonstrated that high motile breast cancer subpopulations with upregulated β integrin subunits were associated with a higher metastatic burden (Desjardins-Lecavalier et al., 2023). Despite high expression of the transcription factors SOX2 and EpCAM in epithelial-like holoclones (Tièche et al., 2019), holoclones showed no extravasation dynamics in our µVN-on-chip system. SOX2 has been reported to be associated with self-renewal capacities and pluripotency, but also invasion and thus poor prognosis in patients. However, amplification and overexpression of SOX2 are associated with more prolonged survival in squamous cell lung cancer (Tang et al., 2022). High EpCAM expression, however, has been described as an epithelial marker associated with cancer stemness and tumor initiation capacity (Liu et al., 2022). In accordance with their phenotype, epithelial-like holoclones show significantly slower migration kinetics compared to mesenchymal-like paraclones. Furthermore, mesenchymal-like paraclones exhibited extravasation dynamics in our µVN-on-chip system, likely due to their high expression of CD90, vimentin, and integrin beta-1 (ITGB1) (Tièche et al., 2019). This variability has been observed in human glioma cells, where tumor progression varies based on CD90 expression levels. High CD90 expression promotes glioma progression *in vitro*, while low CD90 expression in glioma cells stimulates angiogenesis through endothelial cells (Zhang et al., 2018). These data support our results where CD90-low holoclones primarily influence vascular integrity, whereas CD90-high paraclones enhance lung cancer progression through their extravasation capability. Berr et al. identified how genetical and pharmacological disruption of vimentin *in vivo* increases survival as vimentin is required for NSCLC progression, and metastasis and protects from ferroptosis (Berr et al., 2023). Elevated ITGB1 has shown its role in transendothelial migration *in vitro* as well as *in vivo* using metastatic human breast cancer and melanoma cancer cells, suggesting a role in the extravasation dynamics of paraclones (Tièche et al., 2019; Chen et al., 2016). We examined the role of paracrine signaling by A549 subpopulations, holoclones and paraclones, when adding cancer-mediated conditioned medium to the µVN. In contrast to locally induced changes in the permeability coefficient, when cancer cells are directly added to the network, no changes were observed in the vascular barrier integrity. Several studies have reported that dysregulated matrix metalloproteinases (MMPs) are involved in tumor progression by degradation of the extracellular matrix (Kaczorowska et al., 2020). Tièche et al. reported higher MMP-2 expression in paraclones compared to holoclones, but higher MMP-7 expression in holoclones (Tièche et al., 2019). MMP-2 can induce the release of transforming growth factor-beta (TGFβ) and the cleavage of interleukin-1β, which are both related to tumor progression in lung cancer (Sounni et al., 2011; Hannocks et al., 2019). MMP-7 secretion by epithelial-like holoclones might lead to the assumption that these cells support the mesenchymal-like paraclones during migration and tissue invasion but do not impair the vascular integrity in our system. Humayun et al. reported in their microfluidic system that cancer-mediated IL-6, IL-8, and MMP3 secretion led to vascular disruption and thus increased extravasation dynamics of breast cancer cells (Humayun et al., 2021). However, our findings suggest that A549 paraclones extravasate by contact-dependent juxtacrine signaling rather than paracrine signaling, as observed in their extravasation model with breast cancer cells. Gene expression analysis of VE-cadherin (Figure 5) after the addition of conditioned medium of A549 subpopulations supported our results and suggested maintenance of vascular integrity. Furthermore, juxtacrine interaction by ubiquitin-specific peptidase 51/programmed death ligand one/integrin beta-1 has been demonstrated by Li et al. in NSCLC to the malignant tumor progression and therapeutic resistance in NSCLC (Li et al., 2023).

### 4.3 VEGF depletion inhibits CTC extravasation

In recent years, VEGF signaling has shown its importance in vascular angiogenesis as well as in cancer progression, such as cancer cell proliferation, migration, and invasiveness (Shaw et al., 2024). Follain et al. reported that circulating tumor cells used endothelial remodeling to extravasate and that the pharmacological inhibition of VEGF receptor tyrosine kinase could prevent extravasation in zebra fish (Follain et al., 2021). Here we investigated the impact of VEGF on A549 paraclones extravasation capacity. Our results demonstrate that depletion of VEGF in the cell culture medium is sufficient to prevent extravasation while maintaining vessel integrity. Drugs targeting VEGF signaling, such as Bevacizumab (Avastin^®^), have shown promising results in cancer therapies (Garcia et al., 2020; Varlotto et al., 2022). However, VEGF plays a critical role in physiological angiogenesis and thus limits its application *in vivo*. Therefore, combining VEGF-targeting therapies with other drugs is crucial for improving patient outcomes.

Microfluidic-based vascular metastasis models, used in our extravasation assays, enhance our understanding of cancer-specific mechanisms and might serve as potential prognostic tools for cancer therapeutics (Ayuso et al., 2022). Therefore, identifying patient-specific cancer cell dynamics and drug responses will facilitate the development of personalized cancer treatment strategies. ^62,63^. Identifying patient-specific cancer cell dynamics and drug responses will facilitate the development of personalized cancer treatment strategies (Son et al., 2017; Wu et al., 2021; Guenat et al., 2020). Thus, one might imagine sampling cancer cells from patients during tumor resection or biopsies to evaluate the potential metastatic risk of each tumor.

## Data Availability

The raw data supporting the conclusions of this article will be made available by the authors, without undue reservation.
