# Supplementary material for: Assessing the metastatic potential of circulating tumor cells using an organ-on-chip model
Source: Front Bioeng Biotechnol. 2024 Oct 8;12:1457884. doi: 10.3389/fbioe.2024.1457884 (PMC11493642; doi:10.3389/fbioe.2024.1457884)
Supplement: Supplementary file 3 [file DataSheet1.PDF]

# *Supplementary Material*

## 1 Supplementary Figures and Tables

### 1.1 Supplementary Table

**Table S1 List of primer sequences used for RT-qPCR**

| Gene name | Gene sequence (5' – 3') |
|---|---|
| ACTB forward<br>ACTB reverse | GACGACATGGAGAAAATCTG<br>ATGATCTGGGTCATCTTCTC |
| GAPDH forward<br>GAPDH reverse | TCGGAGTCAACGGATTTG<br>CAACAATATCCACTTTACCAGAG |
| HPRT1 forward<br>HPRT1 reverse | ATAAGCCAGACTTTGTTGG<br>ATAGGACTCCAGATGTTTCC |
| CDH5 forward<br>CDH5 reverse | CGCAATAGACAAGGACATAAC<br>TATCGTGATTATCCGTGAGG |

**Table S2 Quantitative analysis of A549 cancer cell extravasation.** The number of A549 holoclones and paraclones was assessed after confocal imaging of the cancer cells in the µVN-on-chip. Images were reconstructed in 3D to quantify extravasated cancer cells. No A549 holoclones were found to extravasate into the surrounding hydrogel. N = 3 per condition.

| A549 Paraclones | # Extravasated Cells |
|---|---|
| Chip1 | 26 |
| Chip2 | 41 |
| Chip3 | 10 |

**Table S3 Quantitative analysis of A549 paraclones extravasation w/ and w/o VEGF.** The number of A549 paraclones was assessed with and without VEGF depletion. Therefore, confocal imaging of the cancer cells in the µVN-on-chip was performed and images were reconstructed in 3D to quantify extravasated cancer cells. N = 4 per condition.

**w/ VEGF:**

| A549 Paraclones | # Extravasated Cells |
|---|---|
| Chip1 | 8 |
| Chip2 | 5 |
| Chip3 | 6 |
| Chip4 | 9 |

**w/o VEGF:**

| A549 Paraclones | # Extravasated Cells |
|---|---|
| Chip1 | 0 |
| Chip2 | 2 |
| Chip3 | 0 |
| Chip4 | 0 |

## 1.2 Supplementary Figures

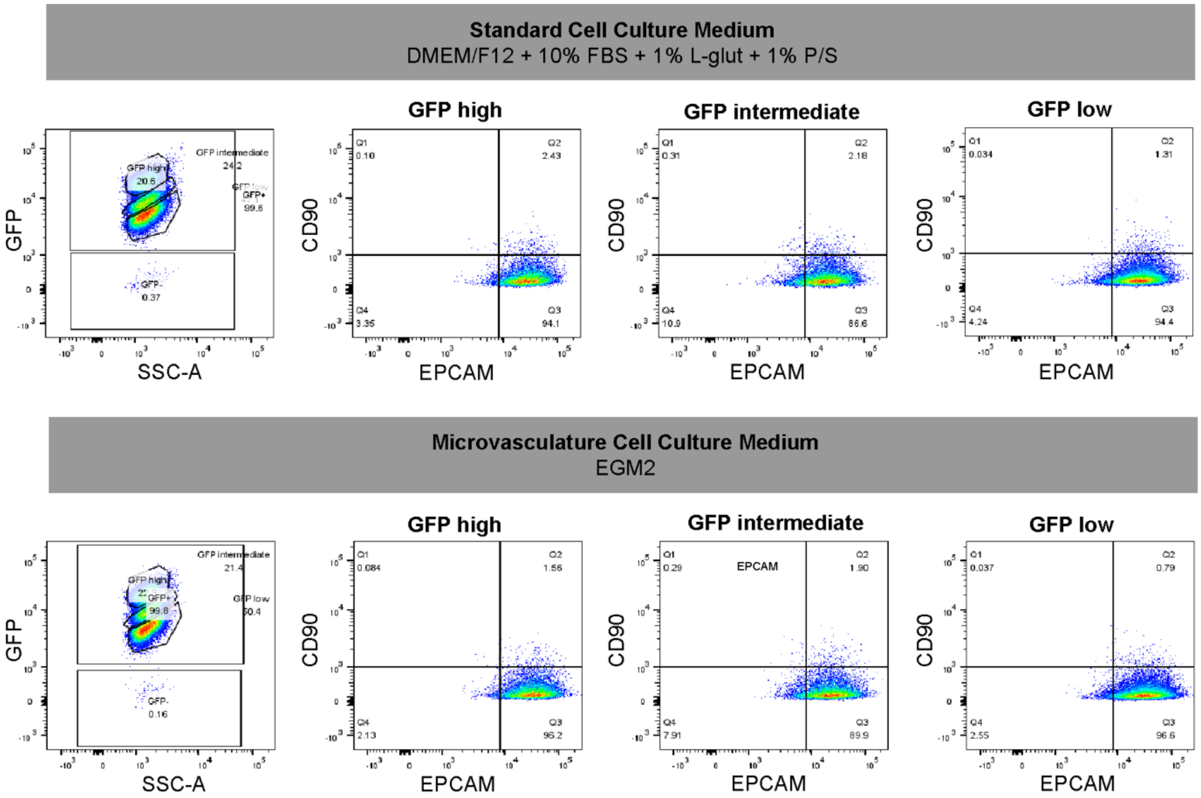


**Supplementary Figure 1. FACS analysis of GFP+ A549 holoclones in standard cell culture medium (DMEM/F12 +++) or microvasculature cell culture medium (EGM2).** A549 holoclones were stained with EpCAM-PE-Cyanine7, mouse, anti-human CD90-BV421 and LIVE/DEAD ™ Fixable Near-IR Dead Cell Stain Kit. Three different subpopulations can be distinguished withing GFP+ holoclones and thus gated by GFP high, intermediate and low signal. All GFP+ holoclones feature a EpCAM+/CD90- phenotype.

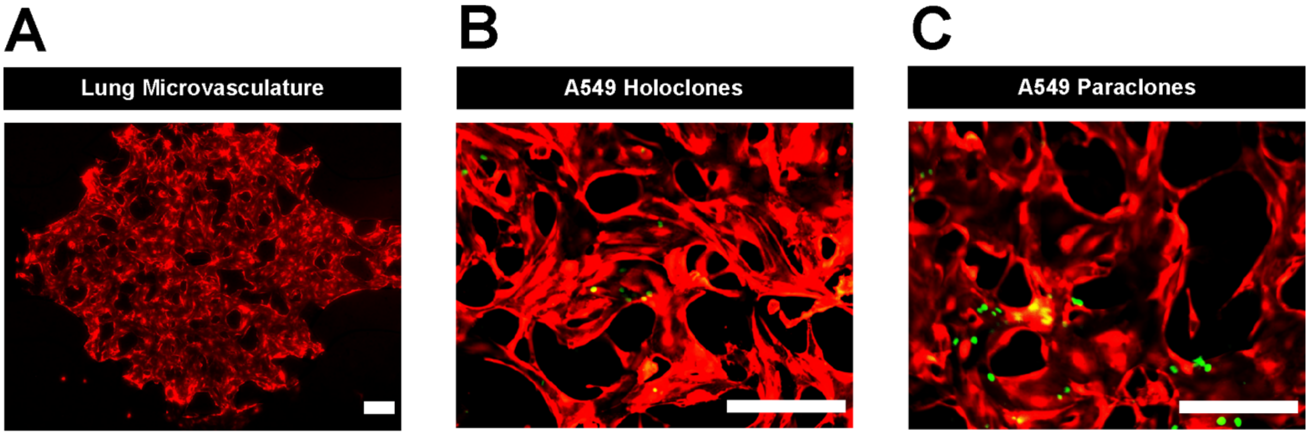


**Supplementary Figure 2. Extravasation assay of two A549 subpopulations in a lung µVN-on-chip.** (A) RFP-labeled primary human lung microvascular endothelial cells (VeraVecs) were co-cultured with NHLFs to form a functional lung microvasculature environment. (B) Representative image of GFP+ A549 holoclones inside the lung microvasculature after 24 hours of incubation. (C) Representative image of extravasating GFP+ A549 paraclones into the surrounding hydrogel. VeraVecs = red, A549 subclones = green, scale bar = 200 µm.

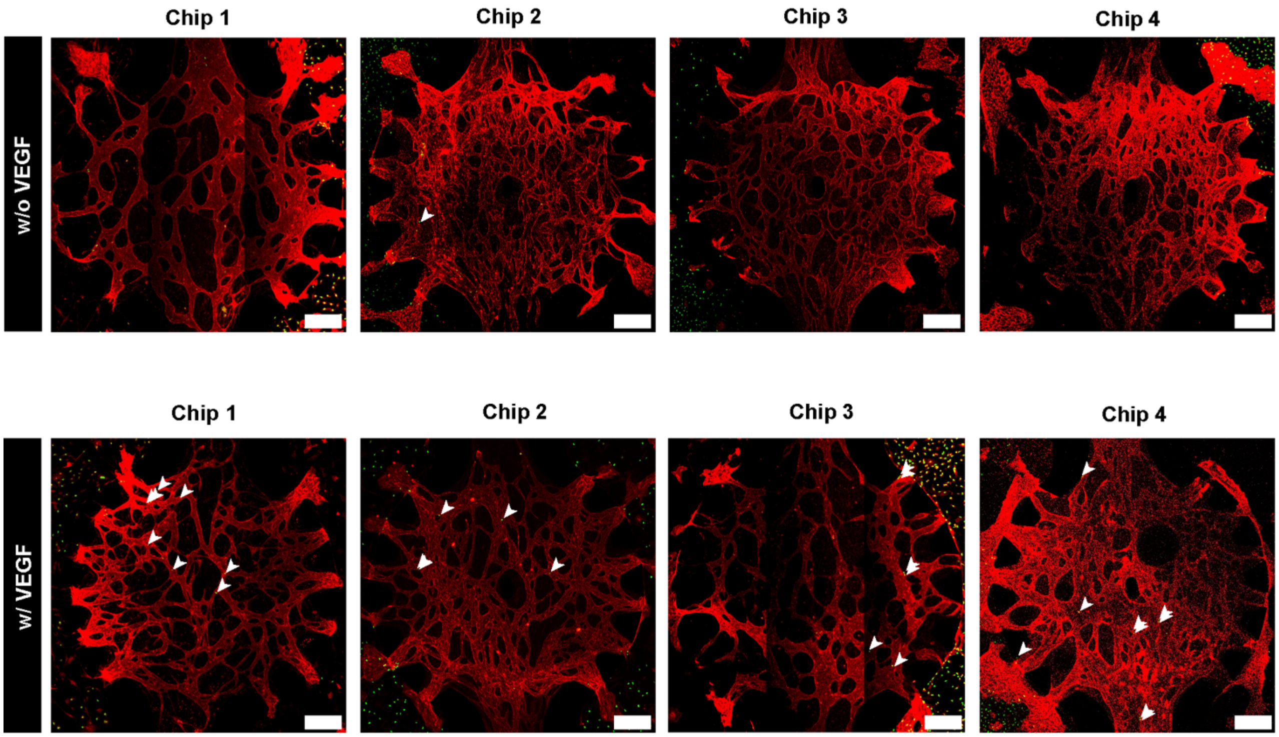


**Supplementary Figure 3. Extravasation assay with or without the presence of VEGF in the microvasculature network.** GFP+ A549 paraclones were added to the microvasculature network after pretreatment of the vasculature on day 5 with (w/) or without (w/o) VEGF in the microvasculature cell culture medium. Endothelial cells were stained with VE-cadherin (red). Immunostaining images show no extravasation dynamics of A549 paraclones w/o VEGF (top row) and extravasation into the hydrogel w/ VEGF (bottom row). Extravasated cancer cells are indicated by an arrow. Scale bar = 200 µm.

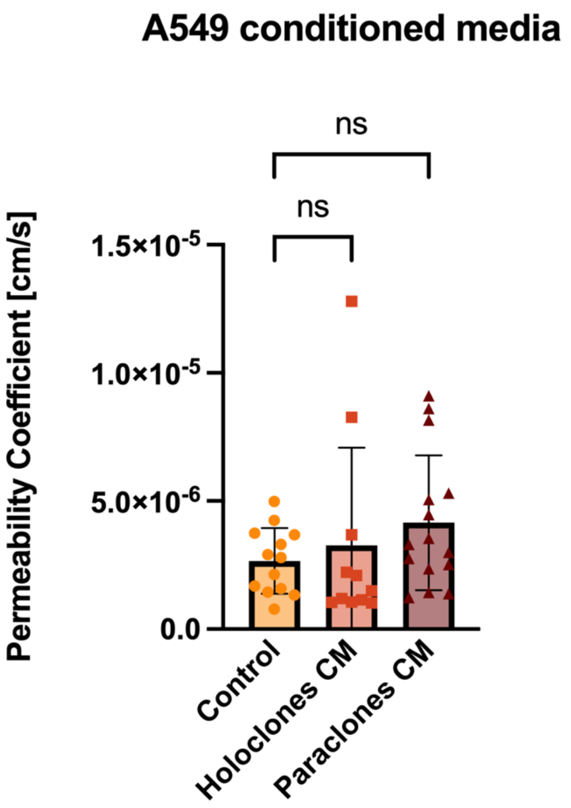


**Supplementary Figure 4. Impact of A549 conditioned medium on vascular permeability.** Conditioned medium of A549 subclones, holoclones and paraclones was added to the µVN-on-chip for 24 hours before the permeability assay. Permeability was assessed with 70 kDa RITC Dextran during three minutes. Plot: mean permeability (SD), statistical analysis: Kruskal-Wallis test followed by Dunn's *post hoc* test for multiple comparisons was used. N = 11-15 chips per condition, at least three independent experiments.

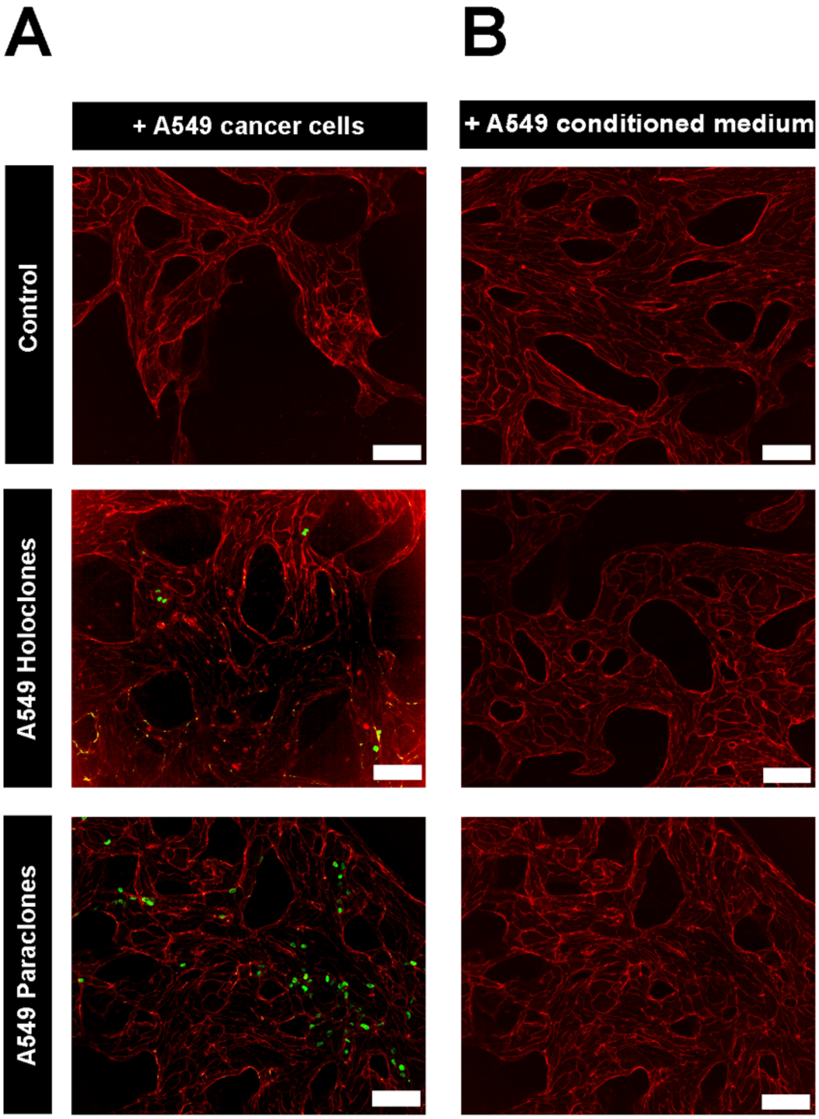


**Supplementary Figure 5. VE-cadherin staining of endothelial cells after exposure to A549 cancer cells and conditioned medium.** The microvasculature network was stained with VE-cadherin (red) after the extravasation assay with [GFP]-A549 holoclones or paraclones (green), respectively, or with A549 conditioned medium. Scale bar = 100 µm.

## 2. List of supplementary videos

| | |
|---|---|
| Video 1 | Microvasculature Permeability Assay with 70 kDa RITC-Dextran |
| Video 2 | 12-hour time-lapse video with A549 paraclones |
| Video 3 | 12-hour time-lapse video with A549 holoclones |
